# Intracardiac Shunt Reversal and Early Right Ventricular Failure after Left Ventricular Assist Device Implantation

**DOI:** 10.7759/cureus.65320

**Published:** 2024-07-24

**Authors:** Ashwin Pillai, Zeina Jedeon, Jonathan Hammond, Jason Gluck, Abhishek Jaiswal

**Affiliations:** 1 Department of Internal Medicine, University of Connecticut Health, Farmington, USA; 2 Department of Cardiology, Hartford Hospital, Hartford, USA; 3 Department of Cardiothoracic Surgery, Hartford Hospital, Hartford, USA; 4 Department of Cardiology/Advanced Heart Failure, Heart Transplant and Pulmonary Hypertension, Hartford Hospital, Hartford, USA

**Keywords:** right ventricular failure, patent foramen ovale closure, patent foramen ovale (pfo), amplatzer occluder, right ventricular assist device, lvad (left ventricular assist device), protek duo, impella 5.5

## Abstract

Right ventricular failure (RVF) is a common complication that occurs after a left ventricular assist device (LVAD) is implanted. We report an interesting case of severe and refractory hypoxia during the early postoperative period after HeartMate3 (HM3) (Abbott Laboratories, Lake Forest, IL) implantation resulting in the unmasking of a right-to-left intracardiac shunt through a patent foramen ovale (PFO), triggered by early RVF. Importantly, the patient had a small left-to-right shunt after receiving a left-sided Impella 5.5 micro-axial pump (Abiomed, Danvers, MA, USA) pre-LVAD implantation. We observed improved hypoxia but worsening RVF after percutaneous PFO closure, necessitating right-sided mechanical circulatory support. We outline potential reasons for the significant PFO-related shunting seen after HM3 implantation, but not after Impella 5.5 placement. Uncertainty exists regarding the approach to a PFO in patients undergoing LVAD implantation. We propose an approach based on existing literature.

## Introduction

Left ventricular assist device (LVAD) implantation frequently leads to right ventricular failure (RVF), which contributes to poor outcomes. The increased right-sided pressures associated with RVF may unmask or reverse an existing intracardiac shunt. This can have clinical implications such as significant hypoxia.

We report an interesting case of severe and refractory hypoxia during the early postoperative period after HeartMate3 (HM3) (Abbott Laboratories, Lake Forest, IL) implantation. The cause of this condition was found to be a new right-to-left intracardiac shunt through a pre-existing patent foramen ovale (PFO), which was probably exacerbated by early RVF. Notably, the patient had a small left-to-right PFO shunt following the Impella 5.5 micro-axial pump (Abiomed, Danvers, MA, USA) before HM3 implantation. We outline potential reasons for the significant trans-PFO shunt reversal seen after HM3 implantation, but not after Impella 5.5 placement. Uncertainty exists regarding the ideal approach to a PFO in patients undergoing LVAD implantation. We propose an approach based on existing literature.

This article was previously presented as a meeting abstract at the International Society for Heart and Lung Transplantation Annual Scientific Conference in Prague, Czechia, on April 12, 2024 [1].

## Case presentation

A 78-year-old male with underlying ischemic cardiomyopathy and atrial fibrillation, who had been hospitalized multiple times for decompensated heart failure, had been transferred to our facility for advanced heart failure management. During his initial admission at our facility, echocardiography revealed a severely reduced left ventricular ejection fraction (LVEF) of 20%, a small left-to-right shunt via a PFO, and severe mitral and tricuspid regurgitations. Right heart catheterization confirmed our clinical suspicion of a low cardiac output state. Initiation of intravenous milrinone improved energy level, walk distance, and tolerance to medical therapy. At the time, the patient declined to undergo an assessment for the placement of a durable LVAD as a permanent treatment. In accordance with his wishes at the time, he was discharged home with inotropic support to improve his quality of life and alleviate his symptoms as part of palliative care.

However, he presented again a month later with clinical cardiogenic shock refractory to escalating supports including the addition of a second inotrope, dobutamine, and the placement of a femoral intraaortic balloon pump (IABP). At this time, he wished to undergo LVAD implantation. The decision to upgrade mechanical circulatory support to the Impella 5.5 was made due to the patient’s inability to wean off inotropes, falling urine output, and inability to tolerate ambulation or bed tilting while on the IABP. This upgrade also helped in promoting pre-LVAD prehabilitation, nutrition, education, and an elective HM3 implantation rather than an urgent one. Impella flow rates were gradually titrated up, carefully monitoring central venous pressures (CVPs) concurrently to ensure right ventricle (RV) tolerance of the increased preload. Absolute flow rates approximated 4.1 L/min at the P8 level of support. Echocardiography performed while on Impella support showed a persistent, small left-to-right shunt across the PFO. There was no hemodynamic or clinical evidence of RVF while being supported with Impella 5.5. Invasive hemodynamic data through sequential therapeutic interventions is shown in Table 1. He had an episode of hemodynamically significant blood loss from gastrointestinal bleeding which improved with blood transfusions although endoscopic evaluation was unrevealing.

**Table 1:** Sequential hemodynamic changes with therapeutic interventions CI: cardiac index; CO: cardiac output; CVP: central venous pressure; IABP: intraaortic balloon pump; mPAP: mean pulmonary arterial pressure; POD: postoperative day; PCWP: pulmonary capillary wedge pressure; SvO2: systemic venous oxygen saturation; SVR: systemic vascular resistance; U: mcg/kg/min; VP: vasopressin; RA: right atrial

Type of Support	RA	mPAP	PCWP	SvO_2_	SVR (dyne/cm^5^)	Fick CO (L/min)	Fick CI (L/min/m^2^)
Home Milrinone 0.25 U	8	22	16	64.9	1890	3.4	1.7
Milrinone 0.375 U + Dobutamine 5 U	11	30	-	66	699	7.1	3.5
Milrinone 0.375 U + Dobutamine 5 U + IABP	9	22	-	62.1	1422	3.6	1.9
Milrinone 0.375 U + Dobutamine 5 U + Impella 5.5	5	37	21	64	1095	4.6	2.4
Pre-DT LVAD	7	31	-	62.6	1294	3.9	2.0
Post-DT LVAD (POD 1) on Milrinone 0.25 U + Epinephrine Drip	12	16	10	54	1319	3.6	1.8
Post-PFO closure on Dobutamine 5 U + Milrinone 0.125 U	12	-	-	-	-	-	-
RV failure on Dobutamine 5 U + Epinephrine and VP Drips + Milrinone 0.125 U	17	22	6	65	1489	3.6	2.2
Protek Duo	6	-	-	63	-	6.2	3.1
Off-Protek Duo + Dobutamine 2.5 U	6	-	-	71.1	-	5.3	2.6
Off inotropes and MCS	4	-	-	58.5	-	4.3	2.2

Subsequently, after 25 days of Impella placement, he underwent implantation of an HM3 LVAD as destination therapy. Intraoperative transesophageal echocardiography (TEE) showed an ejection fraction of 20 to 24% with a normal RV function, severe mitral regurgitation, and a PFO measuring 0.56 cm² with left-to-right shunting. In the immediate postoperative period, he became vasoplegic requiring high doses of vasoactive support to separate from cardiopulmonary bypass.

**Figure 1:**
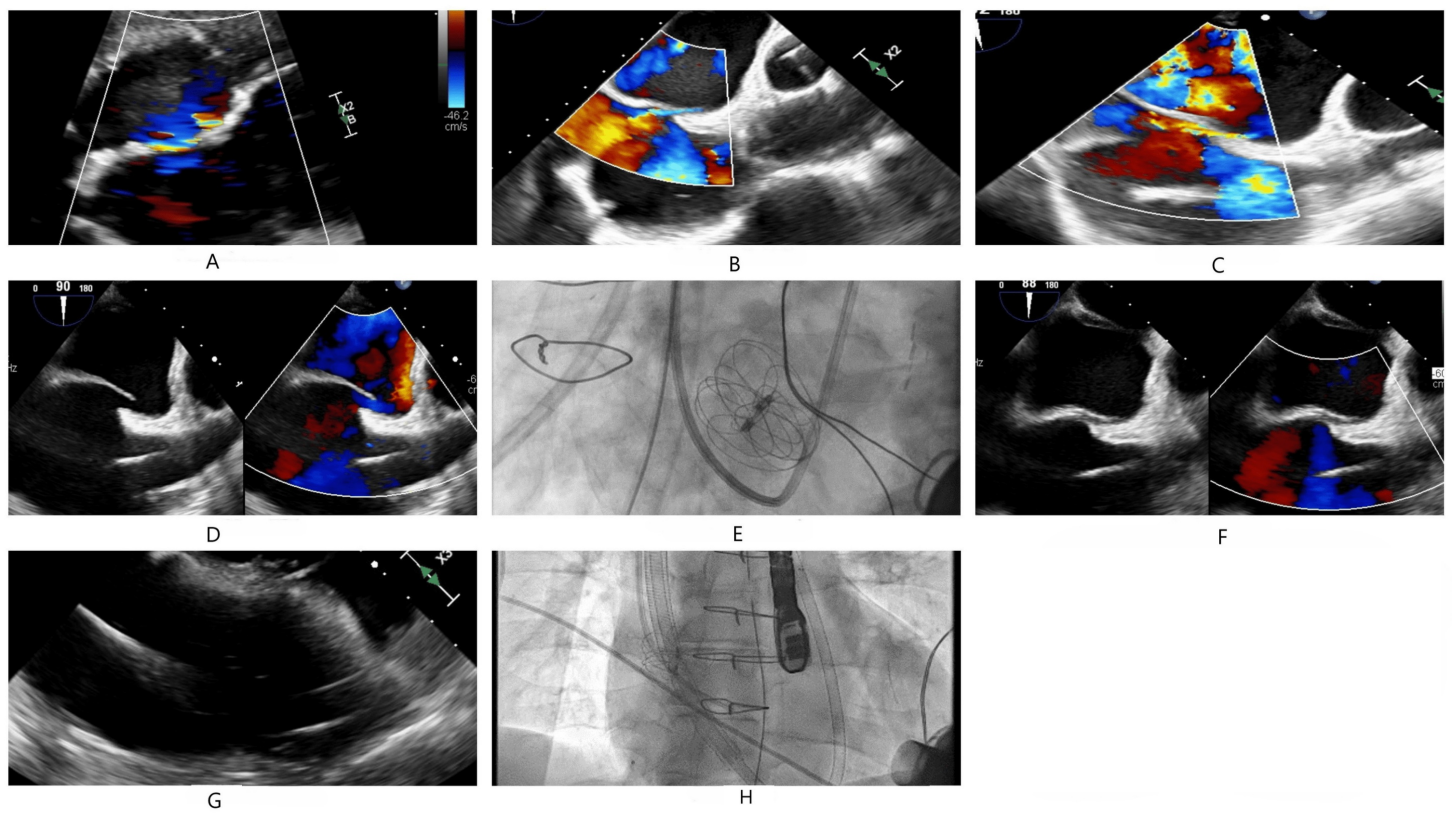
Evolution of the patent foramen ovale shunt with serial interventions A) Pre-intervention TEE demonstrating a left-to-right shunt; B) Pre-intervention TEE demonstrating a left-to-right shunt; C) Post-LVAD TEE demonstrating a reversed right-to-left shunt; D) PFO pre-closure; E) Amplatzer device placement, fluoroscopic view; F) Closed PFO; G) Right ventricular dysfunction; H) Protek Duo right ventricular mechanical support LVAD: left ventricular assist device; PFO: patent foramen ovale; TEE: transesophageal echocardiogram

In the early postoperative period, he was noted to have progressive hypoxemia despite maximal ventilatory support. The worsening hypoxia along with increased LVAD support and low flow alarms raised clinical suspicion of right-to-left shunt reversal from progressive RVF. An emergent bedside TEE confirmed our clinical suspicion. LVAD flows ranged from 4.2-4.6 L/min at a speed of 5000 rpm with a pulsatility index ranging from 2.1 to 2.7 and power ranging from 2.4 to 3.4 Watts. After a multidisciplinary discussion, a decision was made to pursue percutaneous closure of the PFO with an Amplatzer occluder device to improve refractory hypoxia and prevent paradoxical embolization. During this time RV support was deferred given stable LVAD flow and hemodynamics. However, the following day, the patient developed diuretic resistance, new low flow alarms, and increasing inopressors requirement necessitating placement of a Protek Duo (LivaNova PLC, London, UK) device for RV support. This approach resulted in a rapid improvement in hemodynamics, and urine output, and resulted in the weaning of inopressors. Two weeks later, following significant improvement in RVF, the Protek Duo was successfully weaned and removed. Figures 1A-1H represent the evolution of the PFO shunt with serial interventions undertaken in the care of this patient. He was subsequently discharged to short-term rehabilitation after an extended hospitalization approaching nearly two months and was doing well clinically eight months post-LVAD implantation.

## Discussion

LVAD implantation should ideally improve RV function by reducing left-sided filling pressures and right ventricular afterload. LV unloading should, ideally, also help reverse the long-term remodeling of fixed pulmonary hypertension due to left heart failure, further improving RV function. Despite this, around a third of patients supported with an LVAD develop RVF. Predicting RVF can be challenging, as it could be related to different combinations of patient, surgical, and hemodynamic factors [2]. Some centers/clinicians trial a left-sided Impella 5.5 to assess for the development of clinically significant RVF before consideration of durable LVAD implantation in candidates at risk for RVF.

In our case, RVF became apparent only after transitioning from the Impella 5.5 to the HM3. This event could be explained by several potential causes, including differential hemodynamic effects resulting from differential flow rates achieved by the Impella and the HM3 devices. Indeed, right atrial (RA) pressure remained elevated despite higher LVAD flow while RA pressure significantly decreased after Impella placement (Table 1) [3,4]. The higher HM3 flow, adjusted to meet perfusion requirements, likely contributed to an RV preload that exceeded its capacity and led to elevated RA pressures (Table 1). Additionally, the higher HM3 flow likely caused the bowing of the interventricular septum (IVS) toward the LV with consequent RV cavity distortion. This, in turn, would lead to loss of the septal contribution to RV contractility, rendering the RV more susceptible to the increased preload from the higher HM3 output. Alternatively, as the flow rates were only different by 0.5 liters per minute between these devices, there remains a possibility that for the same level of flow, the Impella might be unloading LV differently than an HM3.

Alterations in ventricular interdependence through pericardiotomy and interventricular septal function likely contributed to RV failure after LVAD implantation as well. A lower RV failure rate with a left lateral thoracotomy approach signals that preserving pericardial integrity during LVAD implantation might be protective for the RV [5-7]. Furthermore, post-cardiotomy, the IVS changes from predominantly longitudinal and oblique to predominantly transverse shortening. These alterations make the RV more sensitive to changes in afterload and preload. The leftward shift due to LV decompression after LVAD implantation alongside pericardiotomy-related changes in interventricular contractile dynamics further limits the contribution of the IVS to RV function, putting the RV at a higher risk of failure [8,9].

**Figure 2:**
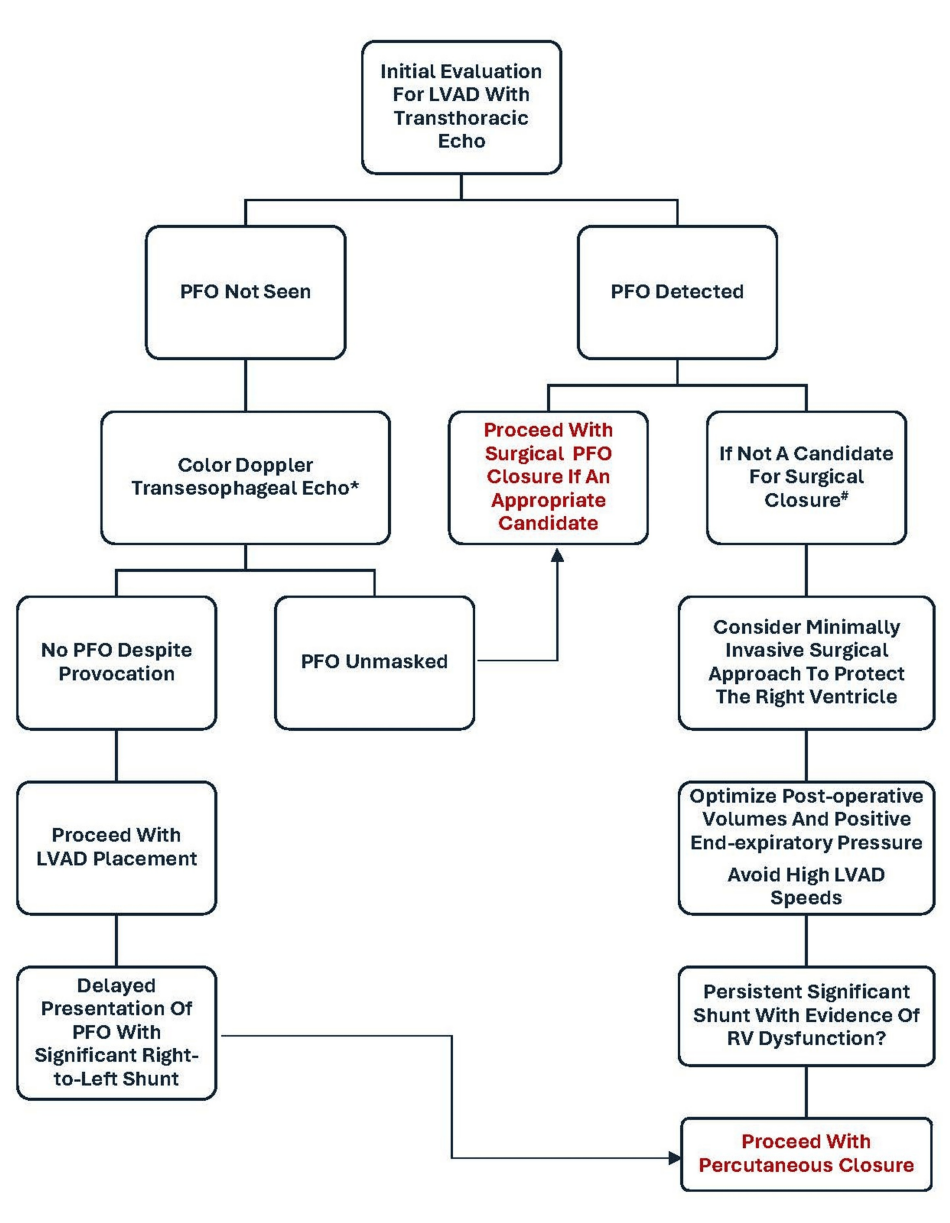
Algorithmic approach to a patent foramen ovale pre-left ventricular assist device placement *May consider transverse pressure to the pulmonary artery to provoke an intracardiac shunt and if present, consider repair ^#^Borderline RV function/ RV dysfunction present/significantly prolonged bypass time LVAD: left ventricular assist device; PFO: patent foramen ovale; RV: right ventricle

The presence of a PFO may elicit undesired consequences in LVAD patients such as hypoxia, like in our case, and has the potential to cause paradoxical embolization and subsequent stroke. The variable physiological status of the patient with heart failure can make pre-LVAD identification and its effects on shunting elusive. When identified, what to do with a PFO remains unclear with some advocating prompt surgical closure. Such an approach while seems intuitive might run the risk of increasing bypass time and risk of RVF in borderline candidates. As such, due to the lack of significant PFO-related shunting after implantation of an Impella 5.5, despite LV unloading and reduced left atrial pressures, we opted to avoid intervention. However, post-LVAD- RVF, PFO-related right-to-left clinically significant shunt warranted management. We demonstrate that percutaneous closure immediately after LVAD is a feasible and safe solution for such events. We summarize a practical approach to PFO in patients who are considered for LVAD implantation as shown in Figure 2.

## Conclusions

In summary, our case highlights the need for thorough exclusion of intracardiac shunts in LVAD candidates, while keeping that the magnitude of the right-to-left shunting could be variable. We highlight that using an Impella might not help in predicting the possibility of PFO shunt reversal post-LVAD due to mechanistic differences between the HM3 and the Impella, as well as post-cardiotomy-related changes in interventricular interactions. Intraoperative TEE can also fail to detect this problem. We also add to the growing body of literature showing the safety and feasibility of percutaneous PFO closure in patients with LVAD as well as the benefits of temporary right ventricular assist device supports in patients with reversible causes of RVF.
